# The Angiogenic Activity of Ascites in the Course of Ovarian Cancer as a Marker of Disease Progression

**DOI:** 10.1155/2014/683757

**Published:** 2014-01-23

**Authors:** Krzysztof Gawrychowski, Grzegorz Szewczyk, Ewa Skopińska-Różewska, Maciej Małecki, Ewa Barcz, Paweł Kamiński, Magdalena Miedzińska-Maciejewska, Wacław Śmiertka, Dariusz Szukiewicz, Piotr Skopiński

**Affiliations:** ^1^Department of Gynecological Oncology and Oncology, Medicover Hospital, Rzeczypospolitej 5, 02-972 Warsaw, Poland; ^2^Department of Obstetrics and Gynecology, Institute of Mother and Child, Kasprzaka 17A, 01-211 Warsaw, Poland; ^3^Department of General and Experimental Pathology, Warsaw Medical University, Pawińskiego 3C, 02-106 Warsaw, Poland; ^4^Department of Pathology, Centre of Biostructure Research, Warsaw Medical University, Chałubińskiego 5, 02-004 Warsaw, Poland; ^5^Department of Applied Pharmacy and Bioengineering, Warsaw Medical University, Warsaw, Poland; ^6^Institute of Mother and Child, Kasprzaka 17A, 01-211 Warsaw, Poland; ^7^1st Department of Obstetrics and Gynecology, Warsaw Medical University, Pl. Starynkiewicza 1, 02-015 Warsaw, Poland; ^8^Department of Surgical Oncology, Holy Family Hospital, Madalińskiego 25, 02-544 Warsaw, Poland; ^9^Department of Oncology, National Institute of Oncology, Wawelska 15, 02-034 Warsaw, Poland; ^10^Department of Histology and Embryology, Centre of Biostructure Research, Warsaw Medical University, Chałubińskiego 5, 02-004 Warsaw, Poland

## Abstract

Ovarian cancer cells are able to create invasive implants in the peritoneum and their growth is directly associated with the angiogenetic potential. This effect is probably stimulated by vascular endothelial growth factor (VEGF) and interleukin-8 (IL-8), which are both found in ascites. The aim of this study was to assess the influence of ascites produced by ovarian cancer on the angiogenesis. Peritoneal fluid was collected from patients with advanced ovarian cancer; cancer cells were separated from CD45+ leukocytes. Angiogenesis was assessed in mice, after intradermal injection of full cellular suspension together with supernatant or phosphate buffered saline, purified cancer cells suspension, or CD45+ leukocytes suspension. The angiogenesis index (AI) was assessed after 72 hours. VEGF and Il-8 were measured in the supernatant and cellular suspension. AI was the highest in the isolated cancer cells suspensions as well in the group stimulated with supernatant. Both VEGF and IL-8 were high in supernatants from ascites rich in cancer cells (>45%). A significant correlation was revealed between IL-8 concentration and AI. We conclude that ascites in patients with advanced ovarian cancer stimulates angiogenesis and this mechanism is dependent mostly on cancer cells activity and enhanced by cooperation with infiltrating leukocytes.

## 1. Introduction

The ovary is a unique organ as it is characterized by physiological angiogenesis. The development of dominant follicle requires a creation of new vessels, ranging from theca surrounding the follicle. The follicle cells, both thecal and granulose, show proangiogenic activity stimulating the migration and proliferation of endothelial cells [1]. During luteinization, endothelial cells migrate from the theca and form capillaries of the dense vascular network to direct most of the ovarian blood flow toward corpus luteum. The basic proangiogenic factor found in follicle fluid is vascular endothelial growth factor-(VEGF-) stimulated by luteinizing hormone (LH), human chorionic gonadotropin (hCG), and basic fibroblasts growth factor (bFGF), as well as interleukin-8 (IL-8). Their role in an ovary is not only the matter of increased angiogenesis but also the stimulation of proliferation of follicle cells and induction of ovulation by increased permeability of follicle vessels [2].

Pathologic angiogenic activity is the attribute of growing neoplasms, when they reach more than 1-2 mm^3^ volume. Numerous proangiogenic factors released by solid tumors as well by tumor infiltrating leukocytes have been described so far [3]. There is no clear evidence which cells are more effective in angiogenesis stimulation. Macrophages isolated from ascites stimulate angiogenesis *in vitro* in chorioallantoic membrane of chicken embryos and release proliferating factor for human umbilical vein endothelial cells (HUVEC) [4]. Cancer cells isolated from ascites show an expression of receptors for bFGF, epidermal growth factor (EGF), and VEGF. Significant concentrations of VEGF are found in ascites; moreover, VEGF seems to be meaningful for production of ascites in the mechanism of increased permeability of peritoneal vessels [5]. The experiments with syngeneic mice showed a correlation between VEGF concentration, amount of cancer cells transplanted, and the volume of ascites. An angiogenic potential of a solid tumor seems to reflect invasive properties of the tumor and to be an important prognostic factor [6]. Different isoforms of VEGF are recognized, depending on an alternative mRNA splicing. VEGF gene consists of 8 exons, where parts 1–5 are always utilized and the combination in splicing of parts 6a, 6b, 7a, 7b, 8a, and 8b gives 15 known isoforms. Exons 6 and 7 encode two independent C-terminal domains, which are responsible for transport of the peptide, signal transduction, heparin affinity, and mitogen activity especially in extracellular matrix [7]. Receptors for VEGF are mostly found on the surface of endothelial cells and they are characterized with tyrosine kinase activity. After binding the ligand, autophosphorylation occurs, followed by signal transduction through cytoplasm with the involvement of phospholipase C (PLC), phosphatidylinositol-3 (PI3) kinase, and tyrosine kinases: c-Src and mitogen activated protein kinase (MAPK) [8].

IL-8 is a dimer represented in four different isoforms—69, 72, 77, or 79 aminoacids—produced mainly by monocytes, lymphocytes T, fibroblasts, endothelial cells, keratinocytes, chondrocytes, and neutrophils [9]. IL-8 is a chemoattractant for neutrophils, lymphocytes T, and basophils, stimulates the release of lysosomal contents, and improves adhering of neutrophils to epithelial cells. IL-8 possess proangiogenic activity as it was proven on HUVEC cells, where IL-8 stimulated a proliferation of these cells. Also, IL-8 stimulated angiogenesis on avascular rabbit cornea models [10]. IL-8 is also involved in growth and maturation of ovarian follicles and blockage of CXCR1/R2 with repertaxin inhibiting the ovulation in murine ovaries [11].

As both VEGF and IL-8 can be found in ascites isolated from patients with ovarian cancer, it seems that ascites can promote angiogenesis within peritoneum, thus promoting growth of invasive implants and progression of the disease.

The aim of the study was the assessment of the influence of ascites produced by advanced ovarian cancer on the angiogenesis in mice.

## 2. Material and Methods

Peritoneal fluid was collected from patients with advanced ovarian cancer either by paracentesis or during laparotomy. The characteristic of the patients is given in Table 1. The patients with histologic type other than serous adenocarcinoma were excluded from the study. The study was reviewed and accepted by the local ethical committee.

### 2.1. Separation of Cells from Peritoneal Fluid

After the collection of the fluid, 50 mL of the fluid was centrifuged (800 rpm, 4°C, 25 minutes); next supernatant was separated and erythrocytes were lysed with different solutions of NaCl. The cells were washed with PBS and counted under light microscope; a cell viability was assessed with trypan blue staining and exceeded 94%.

Lymphocytes and monocytes were separated from cancer cells with magnetic beads coated with anti-CD45 antibodies. Purity of cancer cells suspension was assessed with hematoxylin-eosin staining and exceeded 90%.

### 2.2. Assessment of Skin Angiogenesis

Angiogenesis model was developed in 8-week-old female mice of Balb/c strain, about 20 g of body mass, delivered from the Polish Academy of Sciences breeding colony. Animals were handled according to the Polish law on the protection of animals and (NIH National Institutes of Health) standards. All experiments were accepted and conducted according to ethical guidance of Local Bioethical Committee. Mice were housed 4-5 per cage and maintained under conventional conditions (room temperature 22.5–23.0°C; relative humidity 50–70%; 12 h day/night cycle) with free access to standard rodent diet and water.

After anesthetizing mice with 3.6% chloral hydrate, they were shaved and multiple (2-3) injections were made on each side of the trunk. The following cell suspensions were injected intradermally: full cellular suspension, purified cancer cells suspension, and separated CD45+ leukocytes suspension. Concentrations of cells were equal to 2 × 10^6^ cells/mL; volumes of the injections were 0.1 mL each. The sites of injections were additionally dyed with trypan blue to improve further localization. Next, in mice previously injected with full cellular suspension, angiogenesis was stimulated with subcutaneous injection of supernatant, administered in the neck region after 0, 24, and 48 hours from injection of cellular suspension. In the control group (3 mice for each case), PBS was injected instead of peritoneal fluid. In the 72 hour from initial administration, the mice were treated with a lethal dose of chloral hydrate and skin was separated from subcutaneous tissue in the sites of injections. The formation of new vessels was examined on the inner skin surface, under a dissection microscope at magnification of 32x, and new vessels were counted according to Sidky and Auerbach's method [12]. The angiogenesis index was equal to the mean number of newly formed blood vessels.

### 2.3. Assessment of VEGF and IL-8 Concentration

VEGF and IL-8 concentration were measured separately in peritoneal fluid and cells suspension soon after they were homogenized with ultrasonic probe. Immunoenzymatic method was applied with the use of standard ELISA kit (R&D, USA). Reference concentrations are equal to 15.6 pg/mL, 31 pg/mL, 62.6 pg/mL, 125 pg/mL, 250 pg/mL, 500 pg/mL, and 1000 pg/mL of VEGF and 31.2 pg/mL, 62.5 pg/mL, 125 pg/mL, 250 pg/mL, 500 pg/mL, 1000 pg/mL, 2000 pg/mL of IL-8 were added to the wells coated with anti-VEGF or anti-IL-8 antibody parallel with examined solutions. After incubation in room temperature for 2 hours and rinsing with a producer's buffer, anti-VEGF antibodies conjugated with horseradish peroxidase were added for the next 2 hours and color reaction was achieved after use of hydrogen peroxide and benzidine tetramethyl. The intensity of color reaction was determined in Universal Microplate Reader EL × 800 (Bio-Tek Instruments, Inc., Vermont, USA) at the wave length of 450 nm. The calibration curve was then written with dedicated software and concentration of VEGF and Il-8 was given in pg/mL. The lowest sensitivity of the method was 5 pg/mL for VEGF and 3 pg/mL for IL-8.

### 2.4. Statistical Analysis

Statistical analysis was performed with Statistica 10.0 (StatSoft). Groups were compared with Student's *t*-test and ANOVA with post-hoc test and analysis of correlation was done with Pearson's test. Differences were deemed statistically significant if *P* < 0.05.

## 3. Results

Proportions of cells in ascitic fluid were determined in all patients. There were found: cancer cells (range 0–90%, mean 35.71%), lymphocytes (range 0–95%, mean 37.26%), granulocytes (range 0–55%, mean 5.47%), macrophages (range 0–90%, mean 16.42%), and mesothelial cells (range 0–70%, mean 5.13%).

**Figure 1:**
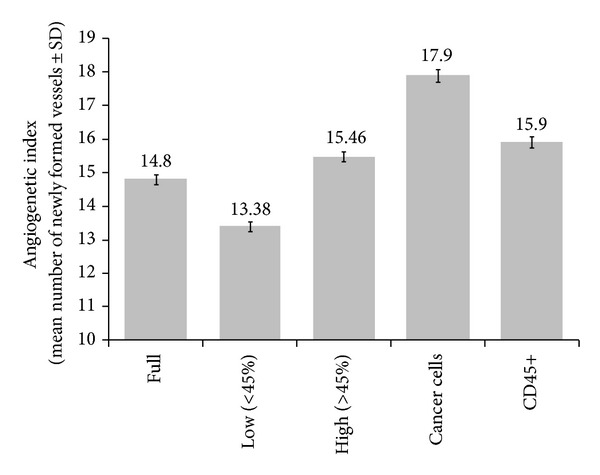
The angiogenetic index was assessed in murine skin after injection with full cellular suspension (Full) and in the subgroup with low (<45%) and high (>45%) percentage of cancer cells (Low), (High), respectively, after injection with separated cancer cells (Cancer Cells) and CD45 positive leukocytes (CD45+). The bars represent mean number of newly formed vessels ±SD. Differences between all groups were tested with ANOVA and were deemed statistically significant.

There was found a significant difference in angiogenesis index between different cellular suspensions; pure cancer cells suspension induced more new vessels than leukocytes CD45+ suspension. Also, full cellular suspension rich with cancer cells (>45%) had high proangiogenic properties. The results are shown on the graph (Figure 1).

After assessment of stimulation of angiogenesis with peritoneal fluid supernatant, it was found that in every case more new vessels were created in the group stimulated with the peritoneal fluid than in control group and the extent of stimulation was not dependent on the amount of cancer cells in the cellular suspension. A total number of 83 sites of neoangiogenesis were assessed in the control group versus 77 in the group stimulated with supernatant, which revealed 14.8 new vessels/site ±0.95 versus 17.1 ± 1.21, respectively. (mean ± SD, *P* < 0.05, compared with student's t-test).

Both VEGF and IL-8 concentration were found significantly higher in supernatants from peritoneal fluid rich in cancer cells (>45%), while in homogenized cell suspension only IL-8 was elevated in the fluid with cancer cells concentration above 45%. The results are shown in Table 2.

The correlation between VEGF and IL-8 concentration and angiogenesis index was analyzed with Pearson's test (Table 3). The positive correlation between IL-8 and angiogenesis index was revealed in patients who received the last chemotherapy dose more than 4 weeks before the ascites collection or did not receive chemotherapy for any time before collection of ascites.

## 4. Discussion

Ovarian cancer still remains one of the most difficult oncologic challenges, as this is diagnosed usually in advanced stage, when radical treatment is difficult and often impossible. Advanced ovarian cancer is characterized with intraperitoneal dissemination and presence of invasive peritoneal implants. Early researches concerning participation of angiogenesis in growth of tumors stated that avascular growth is possible until the tumor reaches 1-2 mm in diameter—the equivalent of 10^5^-10^6^ of cancer cells. In the opposition was the work of Li et al. [13] who showed that even 20–50 cancer cells with “malignant” angiogenic phenotype are enough to start a formation of new vessels, thus confirming the theory of former genetic determination of invasive potential. The aim of this study was to evaluate the angiogenic activity of peritoneal fluid produced in the course of advanced ovarian cancer. We showed on the animal model that in sites of injections of cells isolated from peritoneal fluid new vessels were formed and this process was stimulated with subcutaneous injections of peritoneal fluid. Some differences were also observed between different groups of cellular suspensions: the greatest angiogenic activity showed pure cancer cell suspensions, also significantly higher angiogenic activity was observed in the group of nonseparated peritoneal fluid cells with high concentration of cancer cells (Figure 1). It should be notified that not only isolated cancer cells but also isolated CD45+ leukocytes showed higher activity than nonseparated cell suspensions. We can state that both ovarian cancer cells and infiltrating leukocytes show angiogenic activity which seems to be decreased if cancer cells are faced to leukocytes. This finding is supported by many researches which prove opposite action between leukocytes and cancer cells [14]. Both fractions (supernatant and cellular fraction) of peritoneal fluid contain significant amount of VEGF and IL-8. This reflects their importance in the process of intraperitoneal angiogenesis by increased migration and proliferation of endothelial cells. IL-8 shows chemotaxic properties for leukocytes, which produce the next proangiogenic peptides, thus creating the cascade of angiogenetic events [15]. Our study is concordant with other authors' findings, who confirm high levels of IL-8 in ascites of ovarian cancer. Both, supernatant and cellular fraction rich in cancer cells showed higher levels of IL-8 than those with less than 45% of cancer cells. Thus, it can be concluded, that ovarian cancer cells are capable of IL-8 synthesis and release [16]. VEGF concentrations was significantly higher in supernatant from peritoneal fluid rich in cancer cells. The role of VEGF is not only limited to an induction of neovascularization but also VEGF significantly increases permeability of blood vessels, thus maintaining production of ascites. Cancer implants in peritoneum, while testing on animal model, induced production of ascites and this was a linear function of implanted cells and VEGF concentration [5].

Patients who response to chemotherapy treatment decrease the production of peritoneal fluid. The observation is concordant with the fact that increased levels of IL-8 in ascites are found in patients before they start chemotherapy treatment [17]. In our study, we found the positive correlation between IL-8 and angiogenetic index in patients who had received a chemotherapy regimen more than 4 weeks before the collection of ascites or had not received any. Patients who had been treated with chemotherapy during last 4 weeks before collection of ascites showed poor percentage of cancer cells in ascites and no significant correlation between IL-8 and angiogenesis index was found. This may indicate that angiogenetic potential of ovarian cancer had been considerably reduced with chemotherapy.

The presence of ascites in ovarian cancer is a bad prognostic factor. The ability of peritoneal fluid to angiogenesis stimulation is probably one of the fundamental issues in pathogenesis of disease progression. Present chemotherapy regimens which include antiangiogenic drugs have been proved to improve a progression free survival in the group of patients with high risk of progression [18, 19]. While ascites has the angiogenetic properties, this should be analyzed if frequent paracentesis of peritoneal fluid could improve the results of treatment.

## Figures and Tables

**Table 1:** Demographic characteristic of patients.

Age	Range 41–96	Mean 59
Staging FIGO	IIc–IV	

Histology	Serous adenocarcinoma	*n* = 35
	Mucinous adenocarcinoma	*n* = 2
	Clear cell carcinoma	*n* = 1
	Endometrial carcinoma	*n* = 2

Surgical treatment	Optimal cytoreduction	*n* = 7
	Residual disease	*n* = 28

Chemotherapy treatment (any before the fluid collection)	Yes	*n* = 28
	No	*n* = 7

Chemotherapy treatment in the last 4 weeks	Yes	*n* = 19
	No	*n* = 16

**Table 2:** VEGF and IL-8 concentration determined in supernatant and homogenized cells suspension separated from peritoneal fluid.

	Supernatant			Homogenized cells suspension		
	Group L Mean ± SD	Group H Mean ± SD	*P* value	Group L Mean ± SD	Group H Mean ± SD	*P* value
VEGF (pg/mL)	8931 ± 2245	21851 ± 5798	0.035	5456 ± 1342	9375 ± 3565	0.08
IL-8 (pg/mL)	992 ± 103	2793 ± 230	0.00043	571 ± 140	40047 ± 13123	0.0085

Group L: low percentage of cancer cells (<45%); group H: high percentage of cancer cells (>45%). The groups were compared with student's *t*-test and deemed statistically significant if *P* < 0.05.

**Table 3:** The correlation indices between angiogenesis index (mean number of newly formed vessels) and VEGF and IL-8 concentration determined in supernatant separated from peritoneal fluid. Description: *treatment* means any chemotherapy received less than 4 weeks before fluid collection and *no treatment* means no chemotherapy or chemotherapy received more than 4 weeks before fluid collection. The correlation indices were estimated with Pearson's test; results statistically significant are in bold.

	VEGF		IL-8	
	Treatment	No treatment	Treatment	No treatment
Angiogenesis index	*r* = 0.354	*r* = 0.239	*r* = −0.0167	**r** = 0.7113
